# Older Adults' Social Stress Profiles: A Cross-Country Comparison of the United States and Mexico

**DOI:** 10.1093/geroni/igab046.1522

**Published:** 2021-12-17

**Authors:** Stephanie Wilson, Christina Marini

**Affiliations:** 1 Southern Methodist University, DALLAS, Texas, United States; 2 Adelphi University, Garden City, New York, United States

## Abstract

Older adults face heightened risks for loneliness due to social isolation. Low-quality relationships also fuel loneliness. Because living arrangements and family norms differ between countries, cultural differences may arise in the stress of isolation, loneliness, and difficult relationships. To examine social stress profiles in the US and Mexico, HRS (N=17,878) and MHAS (N=15,001) participants rated their loneliness, whether they lived alone, and relationship quality with their spouse, children, and friends. Five latent classes emerged in both samples: lonely and isolated; lonely with poor relationships; moderately lonely with ambivalent relationships; moderately lonely and unhappily married; and low social stress. Lonely isolation was most common among Americans (23.4%), but least common among Mexicans (14.0%). The highest risks for loneliness coincided with living alone in the US, but with low-quality relationships in Mexico. Results reveal undercurrents of older adults’ social stress that were common to both countries, as well as important cultural differences.

